# *BRAF* and *TERT* promoter mutations in the aggressiveness of papillary thyroid carcinoma: a study of 653 patients

**DOI:** 10.18632/oncotarget.7811

**Published:** 2016-03-01

**Authors:** Langping Jin, Endong Chen, Siyang Dong, Yefeng Cai, Xiangjian Zhang, Yili Zhou, Ruichao Zeng, Fan Yang, Chuanmeng Pan, Yehuan Liu, Weili Wu, Mingzhao Xing, Xiaohua Zhang, Ouchen Wang

**Affiliations:** ^1^ Department of Surgical Oncology, The First Affiliated Hospital of Wenzhou Medical University, Wenzhou, Zhejiang Province 325000, China; ^2^ Department of Surgical Oncology, The Third Affiliated Hospital of Wenzhou Medical University, Wenzhou, Zhejiang Province 325000, China; ^3^ Laboratory for Cellular and Molecular Thyroid Research, Division of Endocrinology, Diabetes and Metabolism, The Johns Hopkins University School of Medicine, Baltimore, Maryland 21287, USA

**Keywords:** papillary thyroid carcinoma, BRAF V600E mutation, TERT promoter mutation, molecular marker, prognosis

## Abstract

The role of telomerase reverse transcriptase (TERT) gene promoter mutations in the aggressiveness of papillary thyroid cancer (PTC) remains to be further investigated. Here we examined the relationship of *TERT* promoter mutations and *BRAF* V600E with the clinicopathological features of PTC in 653 patients. Sanger sequencing of genomic DNA from primary PTC tumors was performed for mutation detection and genotype-clinicopathological correlation of the tumor was analyzed. *BRAF* V600E and *TERT* promoter mutations were found in 63.7% (416 of 653) and 4.1% (27 of 653) of patients, respectively; the latter became 9.8% when only tumors ≥ 1.5 cm were analyzed. *TERT* promoter mutations occurred more frequently in *BRAF* mutation-positive cases compared to wild-type cases, being 5.3% in the former versus 2.1% in the latter (*P* = 0.050). *BRAF* and *TERT* promoter mutations were each significantly associated with high-risk clinicopathological features of PTC, such as old patient age, large tumor size, extrathyroidal invasion, capsular invasion, and advanced disease stages. Coexistence of *BRAF* V600E and *TERT* promoter mutations was particularly associated with high-risk clinicopathological features, as exemplified by extrathyroidal invasion seen in 54.5% (12/22) of patients harboring both mutations versus 9.9% (23/232) of patients harboring neither mutation (*P* < 0.001). Thus, this study, the largest on *TERT* mutation so far, demonstrates a significant role of *BRAF* V600E and *TERT* promoter mutations in the aggressiveness of PTC, which is particularly robust and cooperative when the two mutations coexist. These results, together with previous studies, establish a significant role of these mutations in the aggressiveness of PTC.

## BACKGROUND

Papillary thyroid cancer (PTC) is the most common endocrine malignancy, which constitutes the primary component of the rapid rise in the incidence of thyroid cancer widely seen in recent decades [1, 2]. In general, PTC is an indolent cancer with a high curability and excellent prognosis, but a subgroup of patients have aggressive disease with a poor prognosis. Given this heterogeneity of the disease prognosis, it is often debatable on how to appropriately manage individual cases of PTC when the goal is to optimize the balance between aggressively treating the cancer to prevent disease recurrence and patient mortality and conservatively limiting the treatment extent to reduce the risk of treatment-associated complications [2–4]. To overcome this challenge will rely on improved risk stratifications to more accurately identify the patients who most likely have a poor prognosis. This can be helped with better understanding of the molecular pathogenesis and identification of useful prognostic molecular markers in PTC.

*BRAF* V600E mutation, the most common oncogene in PTC, is such a potential prognostic molecular marker. By aberrantly activating the MAP kinase signaling pathway, this mutation plays an important role in the tumorigenesis of many human cancers, including thyroid cancer [5–7]. Many studies reported a role of *BRAF* V600E mutation in poorer clinicopathological outcomes of PTC [8–13]. There were other studies, however, that failed to demonstrate such a role of *BRAF* mutation and its prognostic usefulness [14–16]. In a previous relatively small study from our group, *BRAF* mutation was found to be associated with poor clinicopathological outcomes even in papillary thyroid microcarcinoma (PTMC) [17]. Clearly, new large studies with robust power are needed to help reconcile this controversy on the role of *BRAF* mutation in PTC.

Two somatic mutations, chr5:1,295,228C > T and chr5:1,295,250C > T (termed here as C228T and C250T, respectively), in the promoter of the gene for telomerase reverse transcriptase (TERT) were found to be associated with poor clinicopathological outcomes of PTC, including aggressive tumor behaviors and increased disease recurrences, making them potentially useful new prognostic molecular makers for PTC [18–22]. TERT is the catalytic subunit of telomerase, which plays an important role in cell immortalization and tumorigenesis. The two *TERT* promoter mutations were shown to be mutually exclusive and able to increase TERT expression [23, 24]. They were also shown to be associated with aggressiveness of other human cancers, such as melanoma, brain tumor and bladder cancer [25–27]. The prevalence of *TERT* promoter mutations in PTC varied between 7.5%–27% in previous studies [18–22, 28–30]. Some studies showed an association between *BRAF* V600E and *TERT* promoter mutations [19, 21, 28]. One study demonstrated that coexistence of the two mutations was associated with the worst clinicopathololgical outcomes of PTC [21]. These recent findings on *TERT* promoter mutations in thyroid cancer are exciting, but they remain to be confirmed and generalized by further and high-power studies, ideally in different ethnic populations.

In recent years, the incidence of thyroid carcinoma in the Eastern China region has been rising rapidly, with a rate well above the national average in China [31–33]. It is not clear what the genetic patterns are in thyroid cancer in this region. In the present study, we used a large cohort of PTC patients from this region to particularly investigate *BRAF* V600E and *TERT* promoter mutations and their role in the clinicopathological outcomes of PTC.

## RESULTS

### Demographic features of patients and *BRAF* V600E and *TERT* promoter mutations in PTC

Among the cohort of 653 patients with PTC, there were 503 (77.0%) female patients. The mean age ± SD of the patients was 46.5 ± 12.4 years. Most of the cases (58.3%) had a tumor size > 1.0 cm and the mean ± SD of the tumor size in the entire cohort was 1.52 ± 0.91 cm. The rest of the demographic information is presented in Supplementary Table 1. Among the entire cohort of the PTC cases, 416 (63.7%) were found to be positive for *BRAF* V600E mutation. Both *TERT* C228T and C250T mutations were found in this cohort of PTC (Supplementary Figure 1) and they were mutually exclusive and collectively found in 27/653 (4.1%) cases. When only tumors ≥ 1.5 cm were analyzed in the present cohort of PTC, the prevalence of *TERT* promoter mutations was 9.8%.

**Figure 1:**
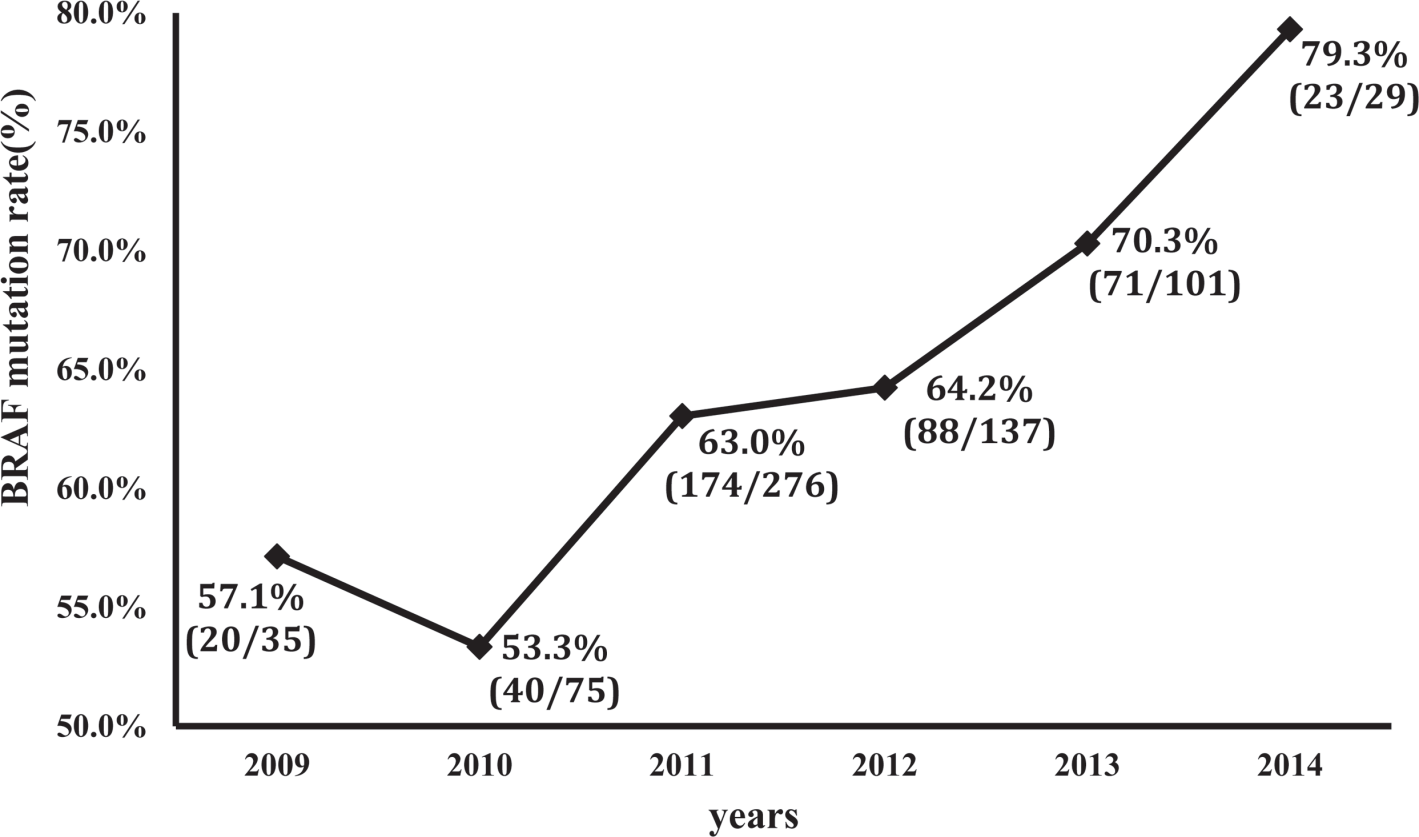
Increasing prevalence of *BRAF* V600E mutation over the time period from 2009 to 2014 The indicated number of papillary thyroid cancer tumors were tested for *BRAF* V600E mutation from the indicted years. Percentages represent the prevalence of *BRAF* mutation. In the brackets, the denominator represents the total cases tested and numerator represents the cases positive for *BRAF* mutation.

**Figure 2:**
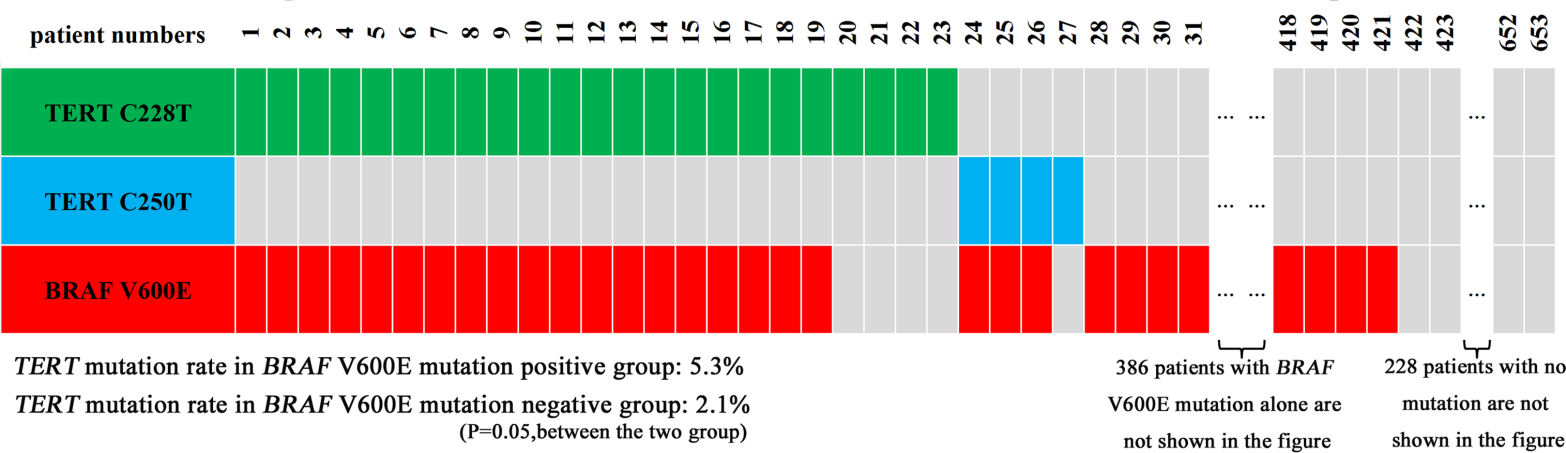
Distribution of *BRAF* V600E and *TERT* promoter C228T and C250T mutations and their relationship in papillary thyroid cancer A total of 653 cases of papillary thyroid cancer were tested for the indicated mutations. Shown are the case numbers and the corresponding mutation status. There was no overlap between the two *TERT* promoter mutations but there was a considerable overlap between *BRAF* V600E and the *TERT* promoter mutations.

Our PTC cases were included from 2009 to 2014. As shown in Figure 1, there was an interesting increase in the prevalence of *BRAF* mutation from the early to the recent years in this period of time. As shown in Figure 2, *TERT* C228T and C250T were mutually exclusive from each other, with the former being dominant, and they were both associated with *BRAF* V600E mutation. Specifically, among the 27 cases that were positive for *TERT* promoter mutations, 22 (81.5%) were also positive for *BRAF* mutation and the prevalence of *TERT* promoter mutations were 5.3% in the *BRAF* mutation-positive group versus 2.1% in the wild-type *BRAF* group (*P* = 0.05).

### Relationship of *BRAF* V600E and *TERT* promoter mutations with clinicopathological features of PTC

As shown in Table 1, when the entire cohort of the 653 PTC cases was divided into two genotype groups—a *BRAF* V600E mutation group and a wild-type *BRAF* group, the *BRAF* mutation group was significantly associated with older patient age (*P* = 0.031), larger tumor size (*P* = 0.006), capsular invasion (*P* = 0.027), extrathyroidal invasion (*P* = 0.004), lymph node metastasis (LNM) (*P* = 0.035), advanced stage (*P* = 0.005) and higher MAICS score (*P* < 0.001). Interestingly, Hashimoto's thyroiditis (HT) was more commonly seen in patients with wild-type *BRAF* tumors (*P* < 0.001). There was no significant association between *BRAF* mutation and patient sex, tumor multifocality (Table 1).

**Table 1:** Relationship between *BRAF* V600E mutation and clinicopathological characteristics of papillary thyroid cancer

Characteristics	*BRAF* status, Number (%)		
	Wild-type (*n* = 237)	V600E mutation (*n* = 416)	*P*-value
Age at diagnosis, y			
Mean ± SD	45.1 ± 12.6	47.4 ± 12.2	0.031
< 45 y	115 (48.5)	162 (38.9)	0.017
≥ 45 y	122 (51.5)	254 (61.1)	
Gender			
Female	182 (76.8)	321 (77.2)	0.914
Male	55 (23.2)	95 (22.8)	
Tumor size in mm			
Mean ± SD	1.37 ± 0.78	1.60 ± 0.97	0.006
≤ 1 cm	112 (47.3)	160 (38.5)	0.028
> 1 cm	125 (52.7)	256 (61.5)	
Hashimoto's thyroiditis	116 (48.9)	112 (26.9)	< 0.001
Multifocality	74 (31.2)	129 (31.0)	0.955
Capsular invasion	40 (16.9)	101 (24.3)	0.027
Extrathyroidal invasion	25 (10.5)	80 (19.2)	0.004
Lymph node metastasis	147 (65.3)*	284 (73.4)*	0.035
AJCC disease stage			
I + II	168 (70.9)	249 (59.9)	0.005
III + IV	69 (29.1)	167 (40.1)	
TERT promoter mutation	5 (2.1)	22 (5.3)	0.050
MAICS score	4.3 ± 0.9	4.6 ± 1.0	< 0.001

* The percentage was calculated only in patients with neck dissection.

Similarly, when the entire cohort of the 653 PTC cases was divided into two genotype groups—a *TERT* promoter mutation group and a wild-type *TERT* group, a significant association was observed between the *TERT* promoter mutation group and high-risk clinicopathological features of PTC (Table 2). Specifically, *TERT* promoter mutations were significantly associated with older patient age (*P* < 0.001), larger tumor size (*P* < 0.001), capsular invasion (*P* < 0.001), extrathyroidal invasion (*P* < 0.001), advanced disease stage (*P* = 0.003), and higher MAICS score (*P* < 0.001). No association was observed between *TERT* promoter mutations and patient sex, HT, tumor multifocality and LNM.

**Table 2:** Relationship between *TERT* promoter mutation and clinicopathological characteristics of papillary thyroid cancer

Characteristics	*TERT* mutation status, Number (%)		
	Wild-type (*n* = 626)	Mutation (*n* = 27)	*P*-value
Age at diagnosis, y			
Mean ± SD	46.0 ± 12.0	59.2 ± 13.8	< 0.001
< 45 y	272 (43.5)	5 (18.5)	0.010
≥ 45 y	354 (56.5)	22 (81.5)	
Gender			
Female	484 (77.3)	19 (70.4)	0.401
Male	142 (22.7)	8 (29.6)	
Tumor size in cm			
Mean ± SD	1.46 ± 0.84	2.80 ± 1.52	< 0.001
≤ 1 cm	270 (43.1)	2 (7.4)	< 0.001
> 1 cm	356 (56.9)	25 (92.6)	
Hashimoto's thyroiditis	220 (35.1)	8 (29.6)	0.556
Multifocality	192 (30.7)	11 (40.7)	0.268
Capsular invasion	125 (20.0)	16 (59.3)	< 0.001
Extrathyroidal invasion	91 (14.5)	14 (51.9)	< 0.001
Lymph node metastasis	411 (70.1)*	20 (76.9)*	0.458
AJCC disease stage			
I + II	407 (65.0)	10 (37.0)	0.003
III + IV	219 (35.0)	17 (63.0)	
BRAF V600E mutation	394 (62.9)	22 (81.5)	0.050
MAICS score	4.4 ± 0.9	6.2 ± 1.4	< 0.001

* The percentage was calculated only in patients with neck dissection.

### Relationship of *BRAF* V600E alone or *TERT* promoter mutation alone or their coexistence with clinicopathological characteristics of PTC

As shown in Table 3, compared with the group harboring neither *BRAF* V600E nor *TERT* promoter mutation, the group harboring *BRAF* mutation alone (no *TERT* promoter mutation) had a significant association with a number of high-risk clinicopathological characteristics. Specifically, *BRAF* mutation alone was associated with older patient age (≥ 45 years) (*P* = 0.042), larger tumor size (*P* = 0.032), extrathyroidal invasion (*P* = 0.012), advanced stage (*P* = 0.022), and higher MAICS score (*P* = 0.002). *BRAF* mutation was significantly less common in patients with HT than patients without HT (*P* < 0.001). Compared with the group harboring neither *BRAF* V600E nor *TERT* promoter mutation, the group harboring *TERT* promoter mutation alone (no *BRAF* V600E mutation) had a significant yet modest association with capsular invasion (*P* = 0.035) and higher MAICS score (*P* = 0.040). There was no significant association between *TERT* promoter mutation alone and other clinicopathological characteristics. Strikingly, the group simultaneously harboring both *BRAF* V600E and *TERT* promoter mutations showed a sharply more common association with much higher significance (lower *P* values) for all the above high-risk clinicopathological features. This association of coexistence of *BRAF* V600E and *TERT* promoter mutations with aggressive clinicopathological features of PTC was far more robust than either mutation alone, with a *P* value being < 0.001 in most cases (Table 3).

**Table 3:** Relationship of *BRAF* V600E alone or *TERT* promoter mutation alone or their coexistence with clinicopathological characteristics of papillary thyroid cancer

	No mutation (*n* = 232)	TERT mutation only (*n* = 5)	*P* value	BRAF mutation only (*n* = 394)	*P* value	TERT+ BRAF mutations (*n* = 22)	*P* value
Age at diagnosis, y							
Mean ± SD	44.9 ± 12.3	53.4 ± 23.0	0.361	46.6 ± 11.8	0.104	60.5 ± 11.2	< 0.001
Range	11–78	25–81		18–79		41–80	
≥ 45 y	119 (51.3)	3 (60.0)	1.000	235 (59.6)	0.042	19 (86.4)	0.002
Gender (Female)	178 (76.7)	4 (80.0)	1.000	306 (77.7)	0.786	15 (68.2)	0.370
Tumor size in cm							
Mean ± SD	1.36 ± 0.78	1.80 ± 0.95	0.247	1.52 ± 0.86	0.032	3.03 ± 1.55	< 0.001
Range	0.1–4.3	0.5–2.8		0.3–5.0		0.6–7.0	
> 1 cm	121 (52.2)	4 (80.0)	0.435	235 (59.6)	0.068	21 (95.5)	< 0.001
HT	114 (49.1)	2 (40.0)	1.000	106 (26.9)	< 0.001	6 (27.3)	0.050
Multifocality	74 (31.9)	0 (0.0)	0.328	118 (29.9)	0.610	11 (50.0)	0.085
Capsular invasion	37 (15.9)	3 (60.0)	0.035	88 (22.3)	0.054	13 (59.1)	< 0.001
Extrathyroidal invasion	23 (9.9)	2 (40.0)	0.088	68 (17.3)	0.012	12 (54.5)	< 0.001
LNM	144 (65.5)*	3 (60.0)*	1.000	267 (73.0)*	0.055	17 (81.0)*	0.150
Late stage (III + IV)	68 (29.3)	1 (20.0)	1.000	151 (38.3)	0.022	16 (72.7)	< 0.001
MAICS score	4.3 ± 0.8	5.5 ± 1.7	0.040	4.5 ± 0.9	0.002	6.4 ± 1.3	< 0.001

Footnotes: Data are expressed as number (percentage). Comparisons were performed between the no mutation group and each of the other three groups (TERT promoter mutation only; BRAF mutation only; TERT + BRAF mutation); *P* value refers to the comparison of the no mutation group with the group in the column immediately left to the *P* value column. *The percentage is calculated only in patients with neck dissection. HT, Hashimoto's thyroiditis; LNM: lymph node metastasis.

## DISCUSSION

In this largest study on *TERT* promoter mutation in PTC, we demonstrated a significant role of *TERT* promoter mutations in the development of aggressive clinicopathological features of PTC. This study on a Chinese cohort of PTC patients confirmed the findings in some previous studies in other ethnic populations [18–22] and expanded this newly emerged exciting area of thyroid cancer research. Given the controversies on the role of *BRAF* V600E in clinicopathological aggressiveness of PTC, we also took the advantage of this large new cohort of PTC to further address this issue. We demonstrated a significant role of *BRAF* V600E mutation in the development of aggressive features of PTC, confirming the findings in some previous studies. With the large size, the present study provides new robust evidence helping reconcile the controversies on this role of *BRAF* mutation.

Several previous studies reported an association between *BRAF* V600E mutation and *TERT* promoter mutations in PTC [19, 21, 28]. One previous study demonstrated that coexistence of *BRAF* V600E and *TERT* promoter mutations was even more strongly associated with poor clinicopathological outcomes of PTC [21]. Inconsistent results on this phenomenon, however, were reported by other studies. For example, in several studies the association between *BRAF* V600E and *TERT* promoter mutations was not significant [18, 22, 34]. Another study even reported a significant inverse relationship between *BRAF* V600E and *TERT* promoter mutations in PTC [29]. Data on the clinicopathological significance of coexisting *BRAF* V600E and *TERT* promoter mutations were also inconsistent; two studies showed no particularly aggressive role of coexisting *BRAF* and *TERT* promoter mutations in the pathogenesis of PTC [20, 22]. These studies, however, were all relatively small. Consequently, the number of cases with coexisting *BRAF* and *TERT* promoter mutations in these studies was all particularly small—too small to provide sufficient statistical power. Thus, this is currently an important unresolved issue on the role of *TERT* promoter mutations in thyroid cancer. Resolution of this controversy would rely on studies of large patient cohorts. Indeed, in the present study—the largest on *TERT* promoter mutation so far, we were able to observe a significant association between *BRAF* V600E and *TERT* promoter mutations and demonstrate a robust cooperative role of coexisting *BRAF* V600E and *TERT* promoter mutations in the development of clinicopatholoical aggressiveness of PTC, providing strong new evidence reconciling the controversy. The present findings, together with previous studies [21], thus firmly establish that coexisting *BRAF* V600E and *TERT* promoter mutations represent a unique robust genetic mechanism that identifies the most aggressive group of PTC and thus has an important prognostic value. This role of coexisting *BRAF* V600E and *TERT* promoter mutations in the clinicopathological aggressiveness of PTC is consistent with the hypothesis that *BRAF* V600E mutation, through constitutively activating the MAP kinase pathway, can promote the expression of *TERT* when *TERT* mutations are created and can thus bind with ETS transcription factors, leading to tumorigenesis-promoting cellular activities [23, 24].

A prevalence of 63.7% for *BRAF* V600E mutation in PTC found in this study seemed to be somehow higher than generally reported, which was averaged around 45–50% [35–37]. One possible explanation is the relatively high dietary iodine intake from rich sea foods in the eastern coastal regions in China where the study was conducted. This possibility is consistent with a previous report of the association between *BRAF* V600E mutation and high dietary iodine intake [38]. Another explanation is that the present study included a large number of patients from recent years, in which there was an increasing prevalence of *BRAF* V600E mutation as demonstrated in the present study (Figure 1). This finding is consistent with other similar reports, including the one of Mathur et al. in which the prevalence of *BRAF* mutation was significantly higher in the recent 5 years compared with earlier years [39]. The lower prevalence of *BRAF* mutation in PTC in an early study from our group seems to be also consistent with this idea [17].

In contrary to the high prevalence of *BRAF* V600E mutation, we found a relatively low prevalence of 4.1% for *TERT* promoter mutations compared with other studies [18–22, 28–30]. One explanation is the high proportion of small tumors in our study; the average tumor size was smaller than those in many previous studies and up to 67.2% of our patients had tumor size < 1.5 cm. Since the present study and previous studies demonstrated an association of *TERT* promoter mutations with larger tumor size of PTC, the large number of small tumors included in our study presumably resulted in an overall low prevalence of *TERT* promoter mutations. This idea is strongly supported by the fact that when only tumors ≥ 1.5 cm were analyzed in the present cohort of PTC, the prevalence of *TERT* promoter mutations rose to 9.8%.

Our study also found an interesting inverse association between the *BRAF* V600E mutation and HT; the prevalence of *BRAF* mutation in PTC was significantly lower in patients with coexisting HT compared with those without HT. This is consistent with similar findings in several previous studies [40, 41]. Interestingly, consistent with an aggressive role of *BRAF* mutation in PTC, PTC associated with HT (and hence lower prevalence of *BRAF* mutation) was associated with a better prognosis in these studies. Microenvironment in which the *BRAF* V600E derives pathogenic molecular changes plays an important role in the pathogenesis of PTC (43–45). It remains to be investigated whether this mechanism may play a role in the interplay among HT, *BRAF* V600E, and clinicopathological features of PTC.

## CONCLUSIONS

This is the largest study on *TERT* promoter mutation in PTC, which demonstrates a significant role of *TERT* promoter mutations and their association with *BRAF* V600E as well as their cooperation in driving the clinicopathological aggressiveness of PTC. This study on a large cohort of PTC also provides new and robust evidence demonstrating an aggressive role of *BRAF* V600E mutation in PTC. These results help reconcile some of the controversies on the roles of *BRAF* V600E and *TERT* promoter mutations in the pathogenesis and progression of PTC and establish their unique prognostic values. As such, this study has important clinical and biological implications for PTC.

## METHODS

### Patients and thyroid cancer samples

A total of 653 PTC patients with complete clinical and pathological data were included in this study, who were treated with thyroidectomy at the First and Third Affiliated Hospitals of Wenzhou Medical University (481 and 172 patients, respectively) from May 2009 to July 2014., Most of these patients were long-term residents living in the coastal Eastern China. Of these patients, there were 503 (77.0%) females. With the ethic committee's approval at the First and Third Affiliated Hospital of Wenzhou Medical University and informed patient consent, fresh PTC specimens were collected from 481 patients undergoing thyroidectomy at the First Affiliated Hospital. Samples were snap-frozen in liquid nitrogen immediately after surgical resection and subsequently stored at a −80°C freezer. Formalin fixed and paraffin-embedded PTC tumor specimens, originally from 172 patients at the Third Affiliated Hospital, were collected for DNA extraction. Histopathological slides were reviewed retrospectively for all cases to confirm the histological diagnosis and to ensure abundant cancer content of the tumor by two pathologists. Disease stages were classified according to the American Joint Committee on Cancer (AJCC) staging system. The MACIS prognostic scoring system was used for predicting PTC patients prognosis as previously described [42].

In our study cohort, there were 88 (13.5%) patients with the age > 60 years at the diagnosis, 132 (20.2%) patients with the tumor > 2 cm, and 612 (93.7%) patients with lymph node dissection, of which 279 (45.6%) were N1a and 152 (24.8%) were N1b. For papillary thyroid cancer (PTMC), central lymph node dissection (CLND) was controversial in our institution. Most physicians performed prophylactic CLND and others did not. For non-PTMC, prophylactic CLND was routinely performed. All lateral lymph node dissection was therapeutic. In this study, no lymph node dissection was performed in 41 patients. None of the patients in this study had distant metastasis. As PTC variants (such as conventional, tall-cell or follicular variant) are not routinely specifically defined in our institution, we report all PTC cases here collectively as PTC. None of the patients had distant metastasis.

### DNA extraction and mutation analysis

For fresh thyroid specimens, genomic DNA was extracted by standard phenol-chloroform extraction and ethanol precipitation procedures. For paraffin-embedded specimens, DNA extraction was performed using the QIAamp DNA FFPE Tissue Kit (QIAGEN) according to the manufacturer's instructions. Exon 15 of *BRAF* was amplified by polymerase chain reaction (PCR) using the following primers: 5′-TCATAATGCTTGCTCTGATAGGA-3′ (sense) and 5′-GGCCAAAAATTTAATCAGTGGA-3′ (antisense). The PCR condition included an initial denaturation step at 94°C for 3 min, followed by 35 cycles of 94°C denaturation for 30 s, 55°C annealing for 30 s, 72°C elongation for 1 min and a final elongation step at 72°C for 5 min. A fragment of the *TERT* promoter, containing the C228T and C250T mutation sites, was amplified by PCR using the GC-RICH PCR System (Roche Applied Science) and primers: 5′-CCAGGACCGCGCTTCCCAC-3′ (sense) and 5′-GGGAGCGCGCGGCATCG-3′ (antisense). The PCR condition included an initial denaturation step at 95°C for 3 min, 10 cycles of 95°C denaturation for 30 s, 60°C annealing for 30 s, 72°C elongation for 30 s, followed by 30 cycles of running at 95°C for 30 s, 58°C for 30 s, and 72°C for 35 s and a final elongation step at 72°C for 7 min. The quality of the PCR products was confirmed by 2% agarose gel electrophoresis. The PCR products were sequenced using a Big Dye Terminator v3.1 Cycle Sequencing Kit (Applied Biosystems) on an ABI PRISM 3730XL DNA Analyzer (Applied Biosystems) to identify the mutation. When a mutation was observed, another independent PCR reaction and sequencing analysis were performed to confirm the mutation.

### Statistical analysis

The results are expressed as mean ± SD for continuous data and percentage (%) for categorical data. Mann–Whitney U test was used for comparison of continuous variables. Pearson *χ*^2^ test or Fisher's exact test was used for comparison of categorical variables. The SPSS statistical software (version 19.0) was used for all analyses. A *P* value ≤ 0.05 was considered to be significant.

## SUPPLEMENTARY MATERIALS FIGURE AND TABLE


