# Supplementary material for: BRAF and TERT promoter mutations in the aggressiveness of papillary thyroid carcinoma: a study of 653 patients
Source: Oncotarget. 2016 Mar 1;7(14):18346–55. doi: 10.18632/oncotarget.7811 (PMC4951292; doi:10.18632/oncotarget.7811)
Supplement: Supplementary file 1 [file oncotarget-07-18346-s001.pdf]

BRAF and *TERT* promoter mutations in the aggressiveness of papillary thyroid carcinoma: a study of 653 patients

Supplementary Materials

Supplementary Table 1: Demographic and clinicopathological characteristics of the patients (*n* = 653)

Characteristics	Number (%)
Age at diagnosis, y	
Mean ± SD	46.5 ± 12.4
Range	11–81
< 45 y	277 (42.4)
≥ 45 y	376 (57.6)
Gender	
Female	503 (77.0)
Male	150 (23.0)
Tumor size in cm	
Mean ± SD	1.52 ± 0.91
Range	1–70
≤ 1 cm	272 (41.7)
> 1 cm	381 (58.3)
Hashimoto’s thyroiditis	228 (34.9)
Multifocality	203 (31.1)
Capsular invasion	141 (21.6)
Extrathyroidal invasion	105 (16.1)
Without neck dissection	41 (6.3)
Number of patients with LNs metastasis	431 (70.4)*
Number of resected LNs, Mean ± SD	10.1 ± 8.8
AJCC disease stage	
I + II	417 (63.9)
III + IV	236 (36.1)
BRAF V600E mutation	416 (63.7)
TERT promoter mutation	27 (4.1)

C228T	23 (3.5)
C250T	4 (0.6)
MACIS score	
Mean ± SD	4.5 ± 1.0
Range	3.2–9.3

LNs: lymph node; *The percentage is calculated in 612 patients with neck dissection.

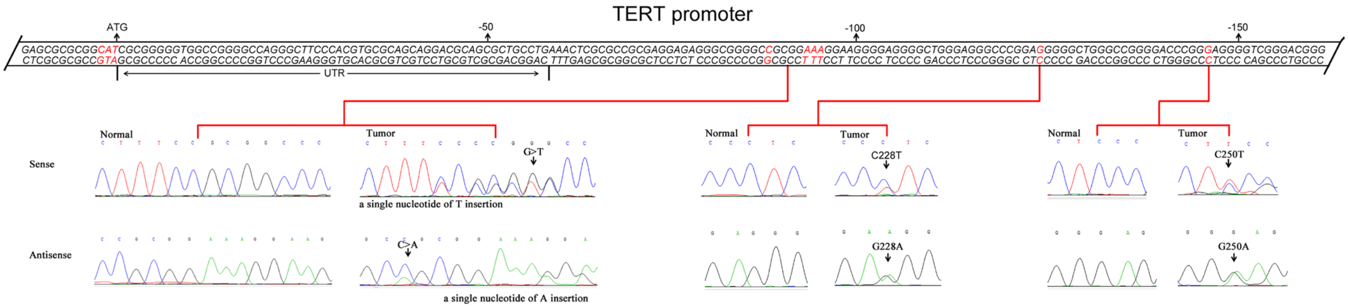

Supplementary Figure S1: Sequencing of the *TERT* promoter in papillary thyroid cancer chromatograms. Shown are the *TERT* promoter C228T and C250T mutations. Shown are also two new *TERT* promoter mutations: a −88G > T mutation and one insert mutation of a single nucleotide between −92 to −96 from the ATG translation start site in one patient.
